# Simple epidemic models with segmentation can be better than complex ones

**DOI:** 10.1371/journal.pone.0262244

**Published:** 2022-01-12

**Authors:** Geon Lee, Se-eun Yoon, Kijung Shin

**Affiliations:** 1 Kim Jaechul Graduate School of AI, KAIST, Daejeon, South Korea; 2 School of Electrical Engineering, KAIST, Daejeon, South Korea; Nanyang Technological University, SINGAPORE

## Abstract

Given a sequence of epidemic events, can a single epidemic model capture its dynamics during the entire period? How should we divide the sequence into segments to better capture the dynamics? Throughout human history, infectious diseases (e.g., the Black Death and COVID-19) have been serious threats. Consequently, understanding and forecasting the evolving patterns of epidemic events are critical for prevention and decision making. To this end, epidemic models based on ordinary differential equations (ODEs), which effectively describe dynamic systems in many fields, have been employed. However, a single epidemic model is not enough to capture long-term dynamics of epidemic events especially when the dynamics heavily depend on external factors (e.g., lockdown and the capability to perform tests). In this work, we demonstrate that properly dividing the event sequence regarding COVID-19 (specifically, the numbers of active cases, recoveries, and deaths) into multiple segments and fitting a simple epidemic model to each segment leads to a better fit with fewer parameters than fitting a complex model to the entire sequence. Moreover, we propose a methodology for balancing the number of segments and the complexity of epidemic models, based on the Minimum Description Length principle. Our methodology is **(a) Automatic**: not requiring any user-defined parameters, **(b) Model-agnostic**: applicable to any ODE-based epidemic models, and **(c) Effective**: effectively describing and forecasting the spread of COVID-19 in 70 countries.

## 1 Introduction

Infectious diseases have been serious threats to global public health. They not only change lifestyles of millions of people worldwide but also bring about dramatic changes in many areas, including economies, cultures, ecologies, and more. Unfortunately, the war against infectious diseases has continued throughout human history. The Black Death killed a third of the world’s population in 1340s, and the Spanish flu in 1918 is estimated to have resulted in at most 500 million deaths. Recent epidemic outbreaks of SARS, Ebola, Zika, and COVID-19 show that the war is not over yet.

Consequently, understanding and predicting epidemic spreads are important for prevention and effective decision making. How many people will be infected within a week? How will lockdowns affect the spread? To answer these questions, we require a method that is simple enough to be comprehensible but expressive enough to accurately model and predict the spread of infectious diseases.

Ordinary differential equations (ODEs) have successfully described dynamic systems in various fields, including ecology, economics, physics, and biology. ODEs have also been utilized in epidemics. Some of the earliest epidemic models, such as SIS, SIR, and SEIR, are compartment models [1]. These models divide the population into several compartments and capture patterns of dynamic changes in the sizes of the compartments over time. The dynamics are expressed as predefined ODEs, which are based on human knowledge, with tunable parameters. While these models are intuitive and simple, they often have limited expressiveness, failing to capture epidemic dynamics accurately. On the other hand, data-driven models [2, 3] aim to model and forecast co-evolving time-series data using ODEs, without relying on human knowledge. They employ latent variables and non-linear differential equations to capture complicated temporal dynamics.

Despite the development of epidemic models, describing long-term dynamics of epidemics using a single epidemic model often faces limitations due to the unpredictability and abruptness of real-world events. Indeed, various external factors may substantially change the dynamics of epidemic events. For example, policies reducing contacts between individuals (e.g., lockdown) and the capability to perform tests can significantly affect the dynamics.

**Fig 1:**
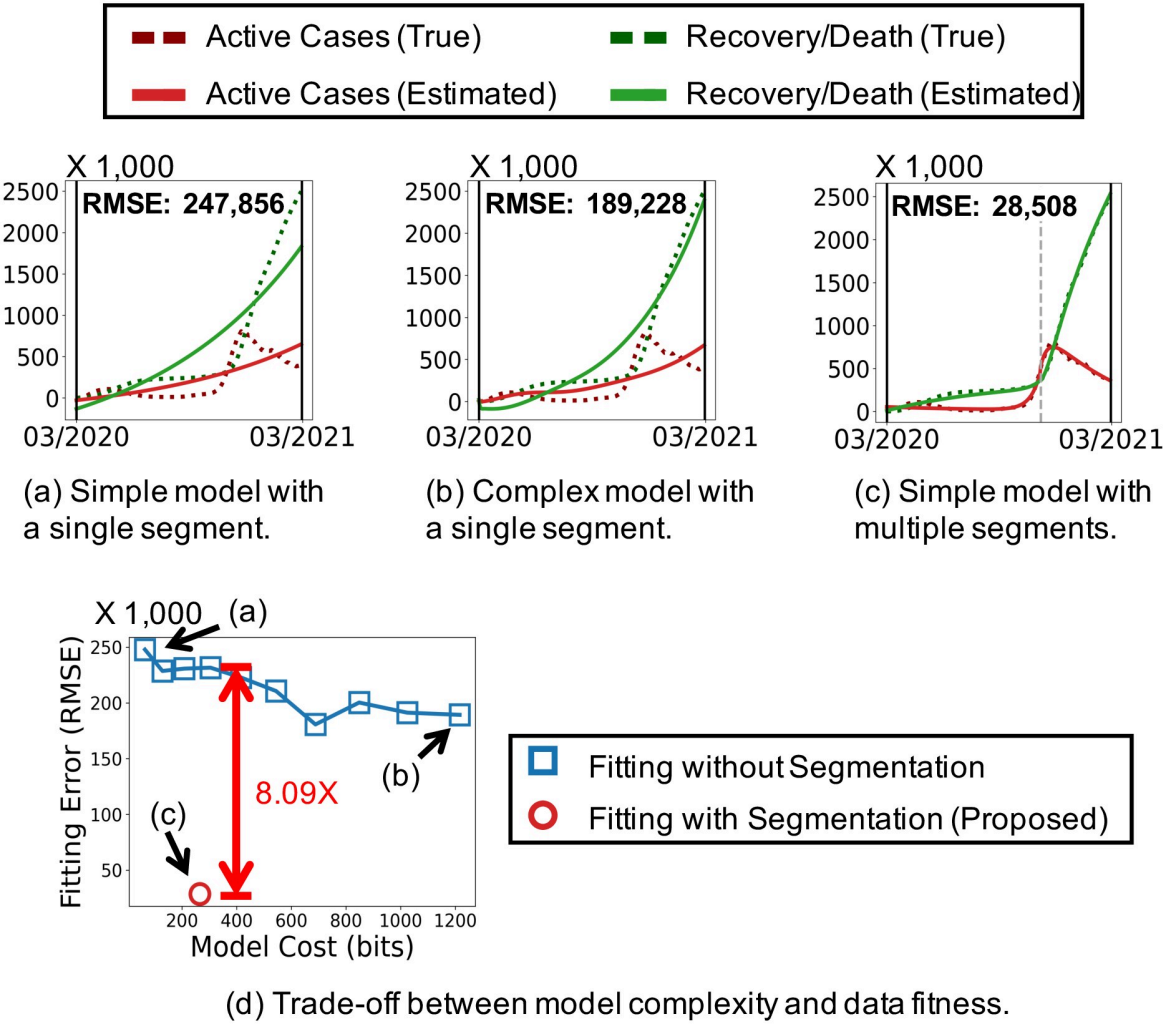
Proper segmentation helps concisely and accurately describe the spread of COVID-19 in Italy. Dividing the event sequence (i.e., the numbers of active cases, recoveries, and deaths) properly into multiple segments and fitting a simple epidemic model to each segment leads to a more concise model with a better fit than fitting a complex model to the entire period. See the experiment section for details.

In this work, we demonstrate that properly dividing an epidemic event sequence into multiple segments and fitting a simple epidemic model to each segment greatly helps describe and predict the epidemic propagation concisely and accurately. For example, in Fig 1(a) and 1(b), the entire sequence of events regarding COVID-19 in Italy is fitted to two epidemic models with different numbers of parameters. On the other hand, in Fig 1(c), the sequence is split into multiple segments, and then a simple model is fitted to each segment. As seen in Fig 1(d), the segmentation leads to 8.09× smaller fitting error with fewer parameters than using a single model for the entire sequence.

Then the following questions naturally arise: Given a sequence of epidemic events, where should we divide it? How many segments should we divide it into? We propose a segmentation scheme that greedily decides where to split. It also decides the number of segments by balancing the fitting error and the sizes of the models for all segments, based on the Minimum Description Length (MDL) principle.

We validate our approach using event sequences regarding recent Coronavirus Disease-19 (COVID-19), specifically the numbers of active cases, recoveries, and deaths in 70 countries. COVID-19 was recognized as a pandemic by the World Health Organization. By early April 2021, 129 million confirmed cases and 2.8 million deaths were reported worldwide. Our experiments reveal that our segmentation scheme enhances three epidemic models in explaining and predicting the propagation of COVID-19.

The strengths of our approach are summarized as follows:

- **Automatic**: It does not require any user-defined parameters, such as the number of segments.
- **Model-agnostic**: It is applicable to any ODE-based epidemic models without being restricted to certain models.
- **Effective**: Applied to the COVID-19 datasets, it significantly reduces the fitting error (up to 14.29× with fewer parameters) and forecasting error (up to 31.54×) of three epidemic models.

Using the proposed segmentation methodology, we expect that real-world surveillance services of COVID-19 can be assisted in following manners:

- **Policy verification**: The point where the segmentation occurs indicates the rapid changes in dynamics, which policymakers should be aware of. Thus, segmentation of the sequence assists examining the impact of policies (e.g., lockdowns or mandatory mask-wearing) after they are deployed.
- **Future prediction**: As shown in the experiments section, future epidemics can be estimated more accurately using our segmentation scheme. Accurate prediction can improve social policy decisions.

**Reproducibility**: The code and datasets used in the paper are available at https://github.com/geonlee0325/covid_segmentation.

## 2 Related work

We briefly review previous work on two related topics: epidemic models and time-series analysis models.

### 2.1 Epidemic models

A variety of epidemic models have been proposed to understand and predict the spread of infectious diseases [4]. In the SI model, the population is divided into two different groups: susceptible and infectious; and the size of each group changes based on predefined differential equations. Taking realistic conditions, such as reinfection, recovery, immunity, population change, and exposure, into consideration, the SI model has been extended to SIS, SIR [5], SIRS [6], SIRD [7], SEIR [8], and many more. The spread of COVID-19 has been analyzed using modified SIRs: Li et al. [9] take human mobility into account, and Dandekar et al. [10] consider quarantine controls. These models are intuitive, explainable, and simple since they are based on human knowledge. However, they show weakness in capturing long-term dynamics of epidemic events especially when the dynamics heavily depend on external factors.

### 2.2 Time-series analysis models

Mining and modeling time-series data is a building block of many analytical and predictive tasks, such as pattern discovery [11, 12], disaggregation [13], and forecasting [2, 3, 14, 15], in a variety of fields, including social media [16, 17], web [14], and medical science [18]. Especially, ordinary differential equations (ODEs) have attracted much attention, due to its simplicity and expressiveness, and several studies focus on learning ODEs from data [19–22]. Recently, Chen et al. [19] introduce a generative model to solve ODEs using neural networks.

There have been several studies on learning to segment temporal data. Most of them [2, 3, 15, 23] focus on detecting repetitive patterns in activities (e.g., sensor data and motion events), while we focus on segmenting epidemic data, where dynamics suddenly change due to external factors, eventually better modeling and forecasting the spread of COVID-19.

Recently, Jiang et al. [24, 25] propose piecewise linear quantile models that detect multiple change-points, where an SN-based test statistic is above the properly chosen threshold, for capturing the ever-changing growth rate of daily new cases of COVID-19. Note that our segmentation scheme has two distinct advantages over those used in these models: (a) automatic: it does not require any prior hyperparameters and (b) model-agnostic: it can be applicable to any ODE-based epidemic models, including non-linear fitting models. Our segmentation scheme belongs to the class of binary segmentation [26]. While existing binary segmentation schemes are known to cause loss when detecting non-monotonic changes [27, 28], we demonstrate that our MDL-based segmentation scheme accurately divides the sequences and fits a model to each segment. Specifically, as shown in the experiment section, our segmentation scheme detects splitting points 3.59× more accurately and leads to 3.23× smaller fitting error (with the same number of parameters) than the non-binary the segmentation method inspired by [2].

## 3 Preliminaries

In this section, we introduce some notations and three main epidemic models that are used in the paper. Refer to Table 1 for the frequently-used notations. We first review the Susceptible-Infectious-Recovered (SIR) model, which is one of the most classical compartment models. Then, we introduce two latent dynamics models that are based on linear and non-linear dynamics of latent variables.

**Table 1:** Frequently-used notations and symbols.

Notation	Definition
*x*(*t*)	observed epidemic event at time *t*
*v*(*t*)	estimated epidemic event at time *t*
*X* = (*x*(1), ⋯, *x*(*n*))	observed epidemic event sequence
*V* = (*v*(1), ⋯, *v*(*n*))	estimated epidemic event sequence
*n*	length of *X*
*d*	dimension of *x*(*t*)
*β*	infection rate
*γ*	recovery rate
*S*(*t*)	susceptible population at time *t*
*I*(*t*)	infected population at time *t*
*R*(*t*)	recovered population at time *t*
*P*	population of the region
*w*(*t*)	latent factors at timestamp *t*
*k*	number of latent factors
*Cost*(*M*)	description cost of model *M*
*Cost*(*X*|*M*)	encoding cost of data *X* given model *M*
*Cost*(*X*)	total cost of *X*
*f*	solver for an epidemic model
*r*	number of segments
$X_{s_{1}:e_{1}}⊕\cdots ⊕X_{s_{r}:e_{r}}$	segmentation of *X* into *r* segments

### 3.1 Susceptible-Infectious-Recovered (SIR) model

The SIR model is one of the most classical epidemic models. Given a group of individuals of closed population *P*, each individual is assigned to one of the three states: *S* (susceptible), *I* (infectious), and *R* (recovered). Here, we use *S*(*t*), *I*(*t*), and *R*(*t*) to denote the number of individuals at the three states, respectively, at timestamp *t*. The model assumes that each individual goes through two types of transitions: infection and recovery. That is, the state to which an individual belongs changes from *S* to *I* and then from *I* to *R*. Additionally, the model assumes that the probability of a susceptible individual to get infected at each time *t* is proportional to the number of infected individuals with a coefficient *β*, and the model assumes that the probability of an infected individual to become recovered at each time *t* is *γ*. These dynamics can be expressed as the following three differential equations, where *β* and *γ* are model parameters:

$$\begin{array}{cc} \frac{dS(t)}{dt} & =-\frac{\beta }{P}\cdot S\left(t\right)I\left(t\right),\\ \frac{dR(t)}{dt} & =\gamma \cdot I(t),\\ \frac{dI(t)}{dt} & =\frac{\beta }{P}\cdot S\left(t\right)I\left(t\right)-\gamma \cdot I\left(t\right). \end{array}$$

Note that these equations imply *S*(*t*) + *I*(*t*) + *R*(*t*) = *P*.

### 3.2 Non-Linear Latent Dynamics (NLLD) model

This model [2] consists of two multi-dimensional event sequences: a *k*-dimensional latent (i.e., unobservable) event sequence *w*(*t*) and a *d*-dimensional observable event sequence *v*(*t*). The observed events *v*(*t*) are assumed to be determined by the following *non-linear* dynamical systems of the latent factors *w*(*t*):

$$\begin{array}{c} \frac{dw(t)}{dt}=p+Q\times w(t)+A⊙(w(t)⊙w(t)), \end{array}$$ (1)

$$\begin{array}{c} v(t)=u+V\times w(t), \end{array}$$ (2)

where ⊙ denotes the Hadamard product (i.e., the elementwise product); and $p\in R^{k}$, $Q\in R^{k\times k}$, and $A\in R^{k}$ describe the linear, exponential, and non-linear dynamics between latent factors. In addition, $u\in R^{d}$ and $V\in R^{k\times d}$ are used to project the latent factors to the observed events. The model parameters are *p*, *Q*, *A*, *u*, *V*, and the initial condition *w*(0) = *w*_0_ of the latent factors.

### 3.3 Linear Latent Dynamics (LLD) model

We also consider a special case of the NLLD model, where the *d*-dimensional observed event sequence *v*(*t*) is assumed to be determined by the following *linear* dynamical systems of *k*-dimensional latent factors *w*(*t*):

$$\begin{array}{cc} \frac{dw(t)}{dt} & =p+Q\times w(t),\\ v(t) & =u+V\times w(t). \end{array}$$

The NLLD and LLD models can naturally be used as epidemic models if we regard *I*(*t*) and *R*(*t*) (i.e., the numbers of infected and recovered individuals) in the SIR model as the 2-dimensional observed event sequence *v*(*t*). Unlike the SIR model, the latent dynamics models are fully data driven, and thus they capture the temporal patterns in the event sequences without any prior knowledge of epidemics. Moreover, they describe the dynamics of the observed events using latent factors, which are not directly observed. Many real-world events are known to be largely affected by latent factors, and as shown in the experiment section, the latent dynamic models predict the spread of COVID-19 significantly more accurate than the SIR model.

#### 3.3.1 Remarks

Our segmentation scheme described in the following section is model agnostic. That is, it can be applied to any epidemic or time-series analysis models, including but not limited to the three considered ones.

## 4 Proposed method

In this section, we present our approach for deciding the number of segments and their locations automatically without user-defined parameters. We first define the description length of an event sequence. Then, based on the definition, we describe how we adapt the Minimum Description Length (MDL) principle to evaluate segmentation. Then, we propose a search algorithm for finding the best segmentation.

### 4.1 Description length

Given a sequence *X* and a model *M*, the description length (in bits) of *X*, denoted by *Cost*(*X*), is defined as:

$$\begin{array}{c} Cost(X)≔Cost(M)+Cost(X|M) \end{array}$$

where the model cost *Cost*(*M*) is the number of bits required to describe the model *M*, and the data cost *Cost*(*X*|*M*) is the number of bits to encode *X* given *M*. The model cost and the data cost are described below.

#### 4.1.1 Model cost

To measure the model cost *Cost*(*M*), we examine the parameters of the model *M* and their sizes in bits. Below, we consider the three aforementioned epidemic models. Note that the model cost of any other models can be measured in a similar way.

- **SIR Model**: The infection rate *β* and the recovery rate *γ* are two real numbers, and encoding each requires *C*_*F*_ bits (we set *C*_*F*_ to 8 by convention). Thus, the model cost required to describe the SIR model in bits is (we ignore the cost required to encode the population *P* since it is required only once regardless of the number of segments):
  $$\begin{array}{c} Cost\left(M\right)=2\cdot C_{F}. \end{array}$$
- **Non-linear Latent Dynamics (NLLD) Model**: This model is described by a set of six parameters: *w*_0_, *p*, *Q*, *A*, *u*, and *V* (see Eqs (1) and (2)). They contain to *k*, *k*, *k*^2^, *k*, *d*, and *kd* real-valued parameters, respectively. Thus, the model cost in bits required to describe the NLLD model is:
  $$\begin{array}{c} Cost\left(M\right)=\left(k^{2}+\left(3+d\right)\cdot k+d\right)\cdot C_{F}. \end{array}$$ (3)
- **Linear Latent Dynamics (LLD) Model**: The model cost required by the LLD model is:
  $$\begin{array}{c} Cost\left(M\right)=\left(k^{2}+\left(2+d\right)\cdot k+d\right)\cdot C_{F}. \end{array}$$
  Note that the cost in bits required to encode *A* is subtracted from Eq (3).

**Algorithm 1**: Segment: MDL-based Greedy Segmentation Search

**Input**: (1) epidemic event stream *X*_1:*n*_

   (2) epidemic model solver *f*

**Output**: segmented event stream $X_{s_{1}:e_{1}}⊕\cdots ⊕X_{s_{r}:e_{r}}$

**1** **if** *n* ≤ 2 **then return** *X*_1:*n*_                 ⊳ Base Case

**2** *C* ← *Cost*(*f*(*X*_1:*n*_)) + *Cost*(*X*_1:*n*_|*f*(*X*_1:*n*_))

**3** $i^{*}\leftarrow \underset{i\in \{2,\cdots ,n-2\}}{\text{arg}\;\text{min}}Cost\left(X_{1:i}⊕X_{i+1:n}\right)$ ⊳ Eq (4)

**4** C* ← *Cost*(*X*_1:i*_ ⊕ *X*_i*+1:*n*_)

**5** **if** C* ≥ *C* **then** **return** *X*_1:*n*_

**6** **else return** Segment(*X*_1:i*_, *f*) ⊕ Segment(*X*_i*+1:*n*_, *f*)     ⊳ Recursive Calls

#### 4.1.2 Data cost

The data cost *Cost*(*X*|*M*) is the number of bits required to describe *X* given *M*. We assume the Huffman coding [29] to encode the difference between the observed event sequence *X* and the event sequence *V* estimated by the model *M*. Then, the number of bits required is the negative log-likelihood under a Gaussian distribution $N(0,\sigma^{2})$ as follows:

$$\begin{array}{cc} Cost(X|M) & =-\text{log}\;P(X-V)\\ & =-\text{log}\prod_{t=1}^{n}\prod_{i=1}^{d}\frac{1}{\sqrt{2\pi \sigma^{2}}}e^{-\frac{{\left(x_{i}\left(t\right)-v_{i}\left(t\right)\right)}^{2}}{2\sigma^{2}}} \end{array}$$

where *x*_*i*_(*t*) and *v*_*i*_(*t*) are the *i*-th dimension of actual and estimated events at time *t*. We fix *σ* to the standard deviation of the elements of *X* − *V* during the period of each segment.

*4.1.2.1 Optimization*. In order to fit *M* to *X*, we use the Levenberg-Marquardt (LM) algorithm to minimize the mean square errors between the given data sequence and the estimated sequence. Specifically, the LM algorithm adaptively varies the parameter updates to be interploated between the Gauss-Newton update or the gradient descent update, by adopting a damping parameter. The lmfit library we used in our implementation requires two arguments xtol and ftol, which are the relative errors desired in the approximation solution and the desired sum-of-squares, respectively. That is, termination occurs (a) when the relative error between two consecutive iterates is at most xtol or (b) when both the actual and predicted relative reductions in the sum of squares are at most ftol. However, as discussed in Section 5.5.1, our segmentation scheme is insensitive to these parameters, and thus we consistently use the same values throughout experiments. For the NLLD model, we split into the linear parameter set (*p*, *Q*, *u*, and *V*) and the non-linear parameter set (*A*) and separately optimize them using the expectation-maximization (EM) algorithm, as suggested in [2]. This, in practice, accelerates convergence, compared to simultaneously optimizing the entire parameters.

### 4.2 Segmentation evaluation

We adapt the Minimum Description Length (MDL) principle [30] for segmentation evaluation. Consider an event sequence *X*(= *X*_1:*n*_) and a solver *f* of an epidemic model. We denote the division of *X* into *r* segments where each *i*-th segment starts at time *s*_*i*_ and ends at time *e*_*i*_ by

$$\begin{array}{c} X_{s_{1}:e_{1}}⊕\cdots ⊕X_{s_{r}:e_{r}}, \end{array}$$

where *s*_1_ = 1, *e*_*r*_ = *n*, and *e*_*i*_ + 1 = *s*_*i*+1_ for each *i* ∈ {1, ⋯, *r* − 1}. Let *f*(*X*_*i*:*j*_) be the epidemic model fitted to the segment *X*_*i*:*j*_. Then, the description length in bits of $X_{s_{1}:e_{1}}⊕\cdots ⊕X_{s_{r}:e_{r}}$ is:

$$\begin{array}{cc} & Cost\left(X_{s_{1}:e_{1}}⊕\cdots ⊕X_{s_{r}:e_{r}}\right)=\left(r-1\right)\cdot {\text{log}}_{2}\left(n\right)\\ & +\sum_{i=1}^{r}(Cost\left(f\left(X_{s_{i}:e_{i}}\right)\right)+Cost\left(X_{s_{i}:e_{i}}|f\left(X_{s_{i}:e_{i}}\right)\right)), \end{array}$$ (4)

where (*r* − 1) ⋅ log_2_(*n*) is the cost in bits required to encode *r* − 1 splitting points (i.e., *s*_2_, ⋯, *s*_*r*_). Since each splitting point is an positive integer smaller than *n*, the number of bits required to encode it is log_2_(*n*). The description length (i.e,. Eq (4)) balances the fitting error and the size of the parameters required to encode the epidemic models for all segments, and we use it to evaluate segmentation. Specifically, based on the MDL principle, we prefer the segmentation that minimizes Eq (4), and in the following subsection, we discuss how we search for such a segmentation.

### 4.3 Segmentation search

Given an event sequence *X*, how can we find the segmentation that minimizes the description length (i.e., Eq (4))? Since there are 2^*n*^ ways to segment a length *n* sequence, naïvely trying all possible segments is computationally prohibitive. Thus, we propose to greedily segment the sequence, as described in Algorithm 1, throughout which we make the length of each segment at least two. Given an event sequence *X*_1:*n*_, we find a splitting point i* ∈ {2, ⋯, *n* − 2} where the description length (i.e., Eq (4)) of the corresponding segmentation is minimized (Line 3). If splitting *X*_1:*n*_ at time i* strictly decreases the description length, we divide *X*_1:*n*_ into *X*_1:i*_ and *X*_i*+1,*n*_, and then recursively divide each segments (Line 6). Otherwise, we stop segmentation (Line 5).

## 5 Experiments

In this section, we review our experiments designed to answer the following questions:

- **Q1. Effectiveness of Segmentation**: Does segmentation help understand the spread of COVID-19? Does it give a better trade-off between model complexity and fitness?
- **Q2. Effectiveness of our Segmentation Scheme**: How well does our greedy segmentation algorithm based on the MDL principle work? Does it yield small fitting error with the same number of segments than baseline?
- **Q3. Accuracy of Forecasting**: Is segmentation beneficial for accurately predicting the spread of COVID-19? Is it beneficial regardless of epidemic models used?

### 5.1 Experimental settings

- **Machines**: We conducted all the experiments on a machine with AMD Ryzen 9 3900X CPU and 128GB RAM.
- **Datasets**: We considered the 70 countries with the most confirmed cases as of the end of March, 2021. We used the number of active cases as *I*(*t*) and the number of recoveries and deaths as *R*(*t*) in each of the 70 countries from March 1, 2020 to March 30, 2021. The dataset is publicly available at [31]. Since the number of recoveries in the US is not available, we used the number of deaths as *R*(*t*).
- **Implementations**: We implemented the SIR model, the LLD model, and the NLLD model in Python. We used the lmfit library for the optimization (see https://lmfit.github.io/lmfit-py/ for details).
- **How to choose *k***: For the LLD and NLLD models, we chose the number of latent factors *k* between 1 and 6 so that the description length (i.e., Eq (4)) is minimized.

### 5.2 Q1. Effectiveness of segmentation

**Fig 2:**
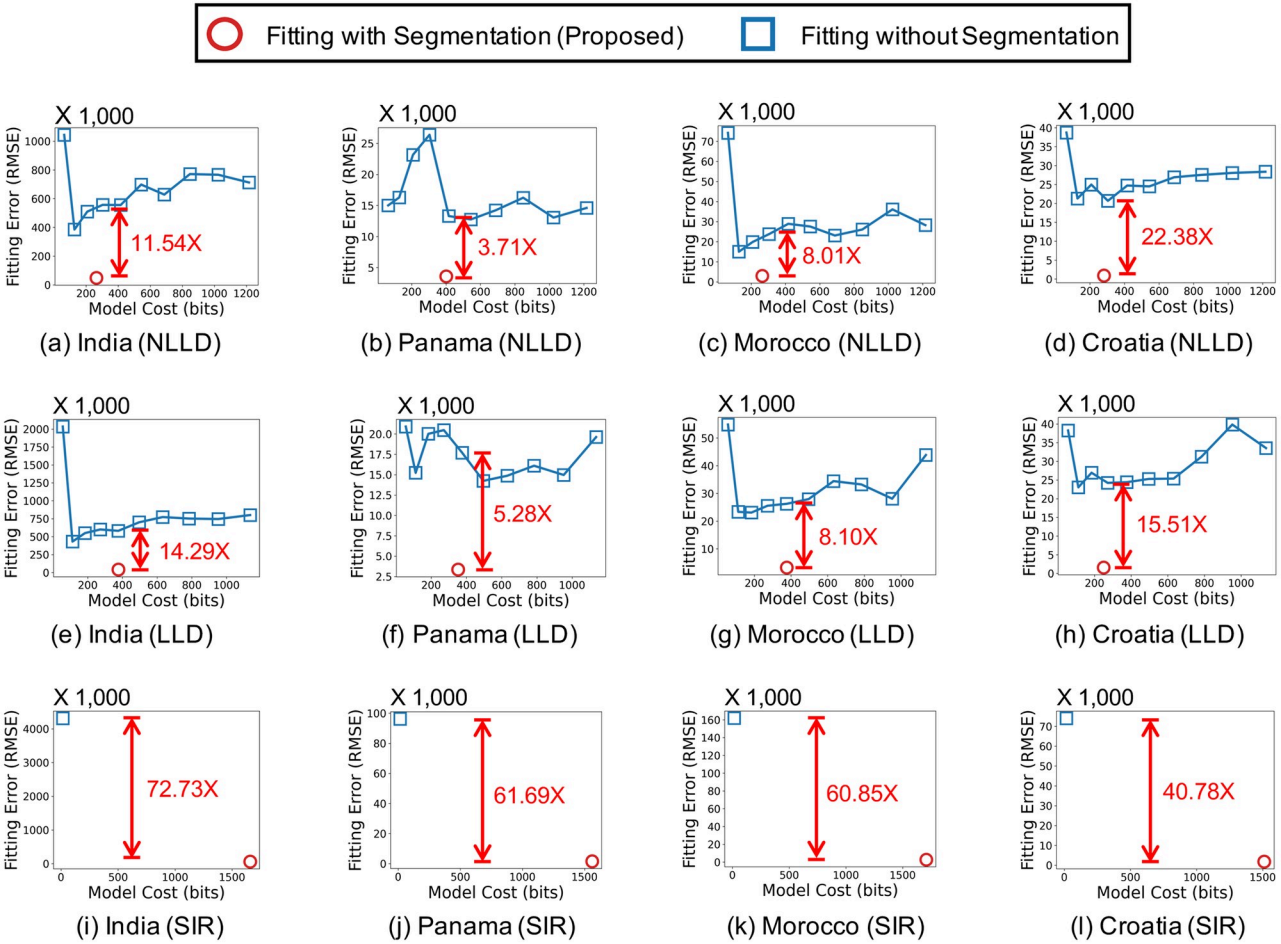
Segmentation leads to better trade-offs between model complexity and fitting error. For the LLD and NLLD models without segmentation, *k* varies from 1 to 10.

**Fig 3:**
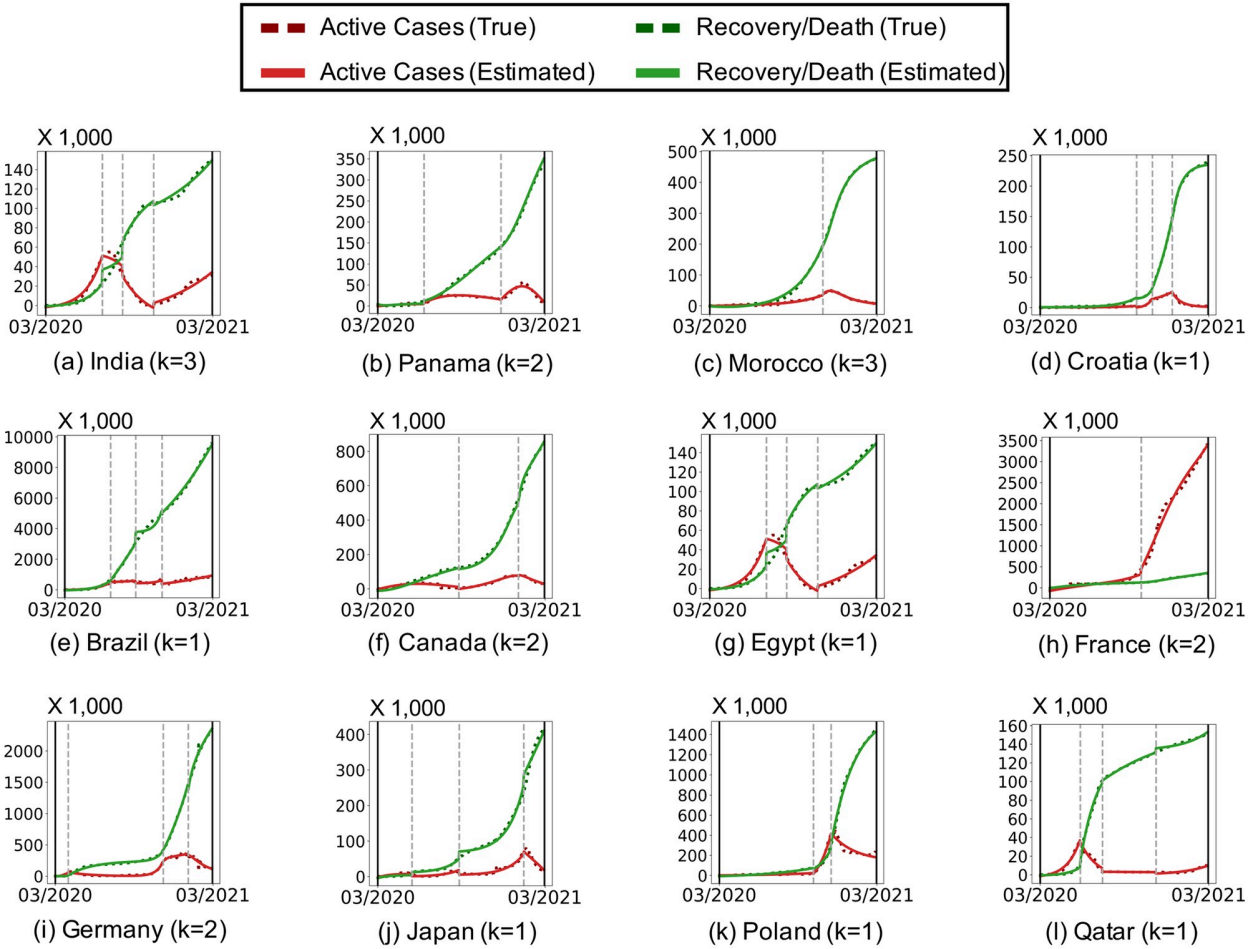
Simple models with multiple segments are preferred over complex models without segments. The true and estimated event sequences when the description length in bits is minimized.

We measure how segmentation by Algorithm 1 affects the model complexity and fitting error of the three considered epidemic models. As seen in Fig 2, segmentation leads to significantly better trade-offs between the model cost (in bits) and the fitting error (in terms of RMSE), regardless of the epidemic models used. For example, in the India dataset, the NLLD model with segmentation yields 11.54× smaller fitting error with smaller model cost than the same model without segmentation. Fig 3 show the input and estimated event sequences when the description length is minimized. The description length is minimized when a simple epidemic model with few latent factors is used with an enough number of segments. **Simple epidemic models with segmentation provide more concise and accurate description of the spread of COVID-19 than complex models without segmentation**. The results in the other countries can be found in the supplement.

**Fig 4:**
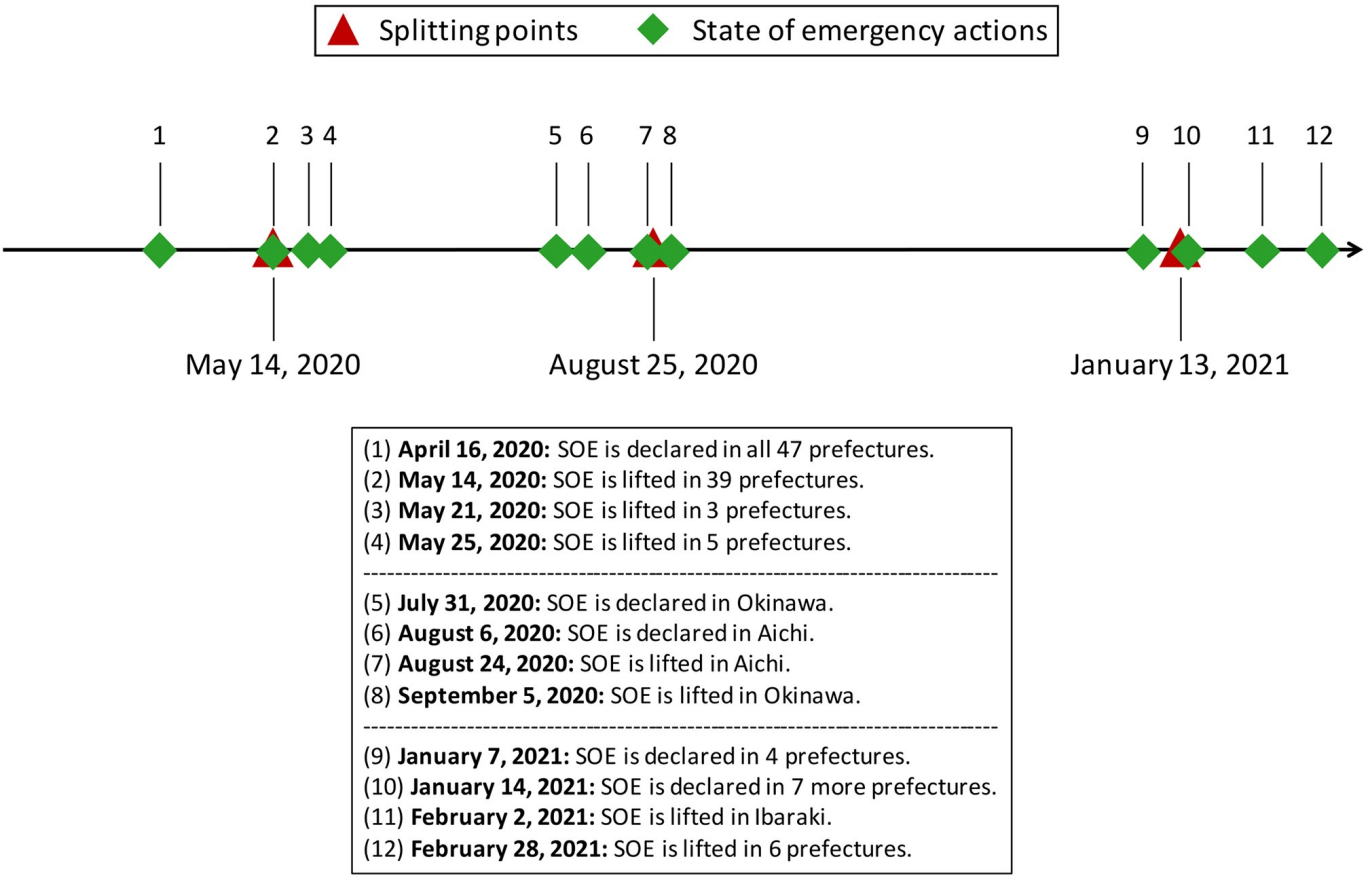
Our proposed segmentation scheme captures policy changes. Splitting points detected by our segmentation scheme coincide with the periods when the state of emergency (SOE) was declared or lifted by the Japanese Government. Note that such events happened 12 times in total during the considered period, and all of them are marked in the figure.

We further qualitatively analyze the splitting points detected by our segmentation scheme in the dataset collected in Japan. Specifically, in the dataset our segmentation scheme detects three splitting points: (1) May 14, 2020, (2) August 25, 2020, and (3) January 13, 2021. As shown in Fig 4, these dates coincide with the periods when the state of emergency (SOE) was declared or lifted by the Japanese Government. The result indicates that there is a close correspondence between the segmentation derived by the proposed scheme and the deployed policies.

### 5.3 Q2. Effectiveness of our segmentation scheme

**Fig 5:**
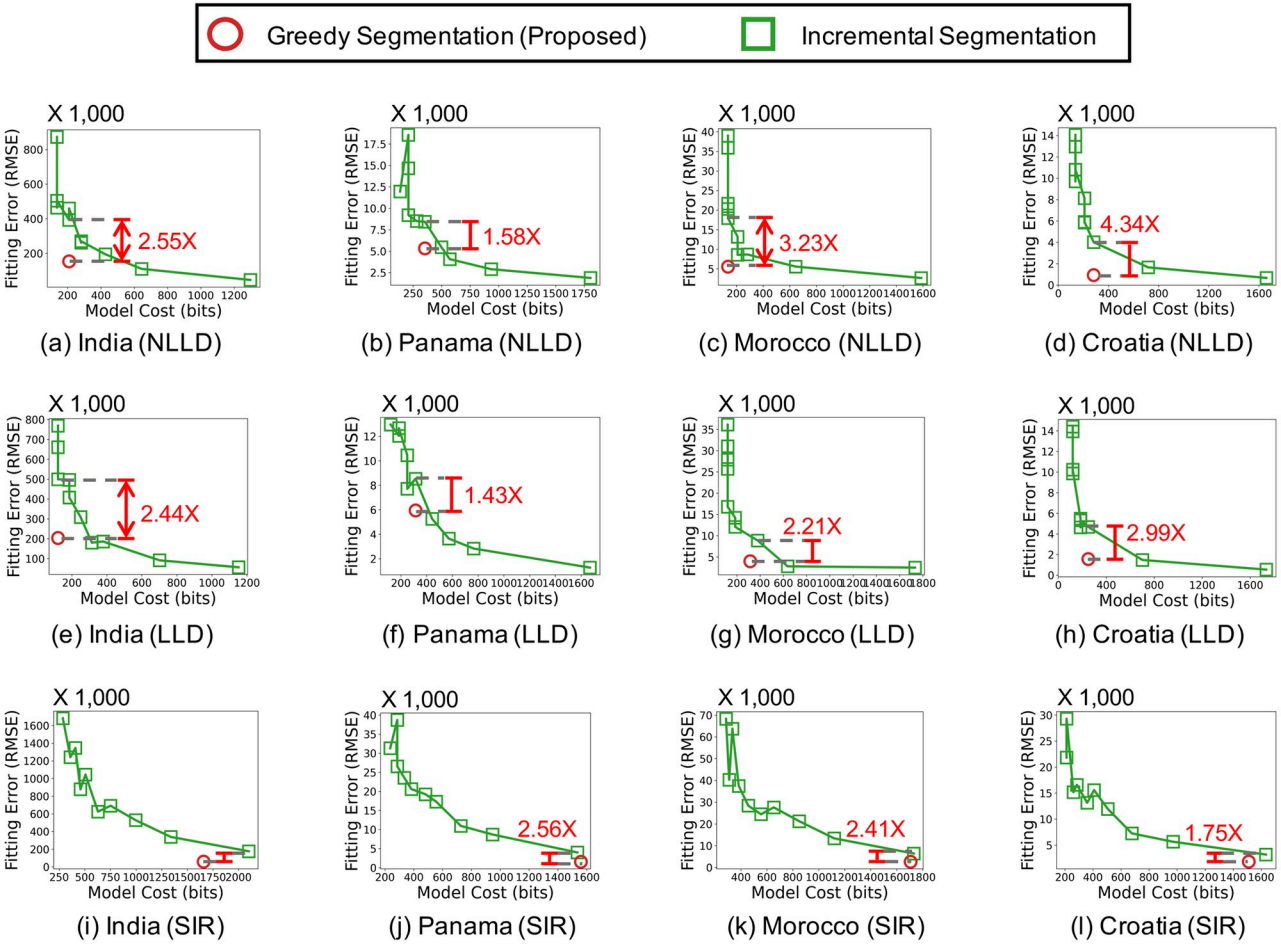
Our proposed greedy segmentation scheme based on the MDL principle yields better segmentation than the incremental method.

We demonstrate the effectiveness of our greedy segmentation scheme based on the MDL principle by comparing it with the incremental method inspired by [2]. The incremental method goes through the sequence from the start and initiates a new segment whenever the fitting error within the current segment exceeds a given threshold *ϵ*. As in [2], we set the threshold proportional to the *L*_2_ norm of the current segment *X*_*c*_ with a coefficient *α*. That is, *ϵ* = *α* ⋅ ||*X*_*c*_||_2_. Note that smaller *α* is expected to yield more segments. As seen in Fig 5, where we fix *k* to 2 and vary *α* from 0.05 to 0.5, our proposed segmentation scheme significantly outperforms the incremental method. Specifically, our scheme gives up to 3.23× smaller fitting error with the same model cost, which is proportional to the number of segments, than the incremental segmentation. The results in the other countries can be found in the supplement.

Furthermore, to numerically evaluate the accuracy of the segmentation, we generate synthetic sequences with randomly selected splitting points where each segment is generated by a different set of random parameters of the NLLD model. We carefully sample parameters based on the model parameters fitted to real-world sequences. Specifically, we sample −0.1 < *p* < 0.1, −0.1 < *Q* < 0.1, −0.001 < *A* < 0.001, −0.1 < *u* < 0.1, −1.0 < *V* < 1.0, and −1 < *w*_0_ < 1 uniformly at random. Then, we compare the detected splitting points, i.e., timestamps where the segmentation occurs, and the ground-truth ones by measuring F1 scores. When measuring F1 scores, for robust evaluation, we consider a detected splitting point is correct if it is within *δ* time units from a ground-truth one. As shown in Table 2, splitting points detected by our segmentation scheme match the ground-truth splitting points closely, and especially, our segmentation scheme is more accurate than the incremental method.

**Table 2:** Our segmentation scheme accurately (in terms of F1 score) detects ground-truth splitting points in synthetic sequences.

Methods	*δ* = 0	*δ* = 1	*δ* = 2
Proposed Method	**0.400**	**0.700**	**0.700**
Incremental Method (*α* = 0.05)	0.000	0.195	0.328
Incremental Method (*α* = 0.10)	0.000	0.100	0.266
Incremental Method (*α* = 0.15)	0.000	0.100	0.100
Incremental Method (*α* = 0.20)	0.000	0.100	0.100
Incremental Method (*α* = 0.25)	0.000	0.000	0.100
Incremental Method (*α* = 0.30)	0.000	0.000	0.100
Random Splitting Method	0.000	0.000	0.000

### 5.4 Q3. Accuracy of forecasting

We examine the effect of segmentation on the the accuracy of future prediction using the three considered epidemic models. To this end, we divide each sequence into the training sequence and the test sequence, which span 327 days and 37 days, respectively. Then, we fit the epidemic models to each training sequence with and without segmentation and predict the event sequence during the test period. When segmentation is applied, we ensure that the last segment is at least as long as the test period, and we use the model fitted to the last segment of the training sequence for prediction. We can ensure this by modifying Algorithm 1 so that it never splits the training sequence during its last 37 days. That is, it searches for splitting points during the first 290 days. This constraint is helpful for forecasting, as shown experimentally in Section 5.5.2. For the LLD and NLLD models without segmentation, we vary the the number of latent factors *k* from 1 to 6.

In Table 3, we compare the prediction error (in terms of RMSE) of the three epidemic models with and without segmentation. When the LLD model or the NLLD model is used, among 7 different settings, our segmentation scheme leads to the most accurate prediction in 32 and 33 (out of 70) countries, respectively. The second best one, which is the LLD model with *k* = 2 and no segmentation, is most accurate only in 9 countries. When the SIR model is used, segmentation increases the prediction accuracy in 70 (out of 70) countries. Moreover, prediction without segmentation is unstable with unreasonably large RMSE in some countries, while it is stable with segmentation in all countries. **To sum up, segmentation tends to improve the prediction accuracy of all three considered epidemic models**.

**Table 3:** Segmentation is helpful to accurate prediction of the spread of COVID-19.

Country	Linear Latent Dynamics (LLD)							Non-linear Latent Dynamics (NLLD)							SIR	
	Single Segment (*r* = 1)						Ours	Single Segment (*r* = 1)						Ours	(*r* = 1)	Ours
	*k* = 1	*k* = 2	*k* = 3	*k* = 4	*k* = 5	*k* = 6		*k* = 1	*k* = 2	*k* = 3	*k* = 4	*k* = 5	*k* = 6			
Argentina	88.2	211.7	**52.4**	53.0	54.5	53.6	56.2	114.0	382.7	329.7	83.7	236.3	**39.6**	65.0	1,301.8	**107.4**
Armenia	43.4	16.3	17.1	16.4	12.8	13.8	**3.7**	38.5	43.0	30.5	10.2	32.6	12.3	**2.2**	115.1	**2.4**
Austria	40.0	45.0	22.8	37.7	20.6	48.6	**10.5**	39.3	74.0	106.7	26.7	68.7	21.1	**10.1**	291.9	**19.3**
Azerbaijan	65.8	125.6	40.2	40.1	40.1	37.4	**11.4**	41.5	37.2	46.8	43.1	40.5	39.7	**12.2**	161.4	**2.6**
Bangladesh	90.3	15.8	10.2	10.2	9.0	11.8	**6.6**	111.8	19.6	27.6	64.0	98.4	**11.7**	27.8	350.2	**6.5**
Belarus	73.4	10.1	10.4	7.8	5.3	5.9	**2.1**	11.5	49.0	73.4	19.9	**6.1**	6.8	6.2	177.7	**24.2**
Belgium	160.0	**16.5**	71.9	67.6	67.0	73.3	21.0	325.1	29.5	85.5	70.8	65.5	38.6	**11.6**	-	**29.4**
Bolivia	14.4	17.3	**3.5**	4.0	5.2	5.7	53.9	9.8	57.7	**3.3**	14.8	4.7	8.5	81.0	133.0	**30.0**
Brazil	289.0	242.8	116.6	120.2	118.5	**115.6**	177.6	223.9	542.7	621.4	193.6	523.3	215.3	**95.0**	6,252.0	**682.3**
Bulgaria	**71.3**	91.4	91.2	77.5	76.2	76.4	78.4	**78.3**	130.4	518.3	126.3	100.5	102.1	89.5	144.4	**13.5**
Canada	65.0	141.2	39.0	120.8	**25.5**	25.5	50.8	84.6	**43.6**	98.5	45.2	61.4	52.0	74.2	545.3	**68.1**
Chile	95.1	211.3	16.2	16.1	16.6	17.7	**13.3**	188.4	29.8	**17.3**	26.7	55.3	111.9	40.9	519.6	**60.9**
Colombia	483.3	94.8	84.8	84.1	82.5	**82.1**	110.0	365.5	602.0	118.0	93.9	**88.9**	97.6	158.3	1,470.1	**161.5**
Costa Rica	23.3	**4.4**	4.5	10.6	12.6	12.9	13.9	37.1	8.4	**6.2**	6.7	9.4	11.8	17.2	117.9	**9.8**
Croatia	61.7	22.3	41.7	26.3	22.6	22.5	**2.8**	104.5	38.5	64.6	32.1	48.6	39.8	**2.4**	164.7	**7.4**
Czech	145.8	**69.0**	71.6	105.7	110.2	109.6	71.8	114.9	172.0	206.8	110.3	**109.6**	111.0	118.1	678.2	**98.9**
Denmark	36.8	22.1	34.2	34.9	33.3	34.7	**11.7**	32.7	32.6	73.1	37.3	37.5	28.4	**19.8**	138.3	**8.8**
Dominican Rep.	9.9	**8.4**	9.8	9.8	9.9	9.3	17.5	11.0	**9.2**	10.8	10.8	9.2	11.2	17.5	127.2	**21.2**
Ecuador	40.0	11.7	10.8	11.0	12.2	11.4	**10.0**	64.3	11.5	11.9	17.1	11.1	11.4	**10.2**	167.2	**19.3**
Egypt	30.4	26.0	**7.7**	8.3	8.0	9.6	14.7	9.7	26.0	9.9	**8.1**	11.4	12.8	13.4	103.0	**8.4**
France	62.8	83.1	76.6	90.3	114.1	95.2	**41.8**	81.8	98.7	159.4	**66.0**	73.3	95.8	92.7	-	**250.4**
Georgia	34.3	26.1	83.4	40.3	29.1	30.1	**15.4**	45.5	64.1	74.1	59.2	38.2	91.4	**6.1**	182.1	**9.5**
Germany	467.4	72.0	71.1	72.5	71.6	72.5	**67.8**	591.2	98.7	180.5	326.0	160.7	104.6	**88.9**	1,513.3	**174.2**
Greece	12.9	14.4	16.3	18.2	17.9	18.7	**12.6**	13.7	16.6	18.8	15.7	18.4	17.9	**10.4**	85.6	**11.7**
Guatemala	38.6	**1.0**	1.0	1.1	1.1	1.3	3.1	32.6	36.3	16.9	**1.0**	23.9	1.1	19.4	110.7	**9.8**
Honduras	**5.7**	8.7	7.9	7.0	8.1	7.2	16.5	**5.1**	11.2	10.5	7.9	7.8	7.2	15.4	78.8	**12.1**
Hungary	119.8	71.0	30.3	**30.1**	30.6	30.4	53.2	168.9	97.7	46.4	34.8	46.7	39.3	**22.9**	218.2	**25.5**
India	3,517.9	145.8	180.3	430.2	590.6	705.5	**45.0**	1,629.2	142.9	278.4	315.5	242.9	424.0	**111.4**	7,571.9	**171.0**
Indonesia	**21.2**	32.7	30.3	23.3	25.7	27.1	45.5	27.3	51.8	28.9	24.9	24.3	**22.7**	70.7	719.7	**162.4**
Iran	271.7	63.5	70.3	70.1	70.4	67.7	**48.7**	182.4	228.1	1,982.8	81.5	76.7	72.5	**65.0**	944.9	**90.9**
Iraq	64.2	50.8	43.1	34.3	**31.7**	35.1	35.3	84.1	53.4	56.0	42.8	35.8	40.1	**27.2**	436.2	**20.6**
Ireland	88.7	55.4	55.3	56.0	17.5	**14.6**	33.7	71.9	76.3	76.4	13.6	13.5	18.0	**2.4**	119.4	**22.3**
Israel	35.3	22.1	21.5	**20.3**	22.1	26.4	131.7	47.4	121.6	19.6	**15.4**	19.9	20.9	87.8	451.3	**92.2**
Italy	459.8	806.5	318.1	362.5	349.1	245.8	**60.0**	550.8	426.8	320.7	321.7	245.8	362.3	**120.7**	1,611.9	**156.4**
Japan	65.1	64.8	**53.9**	54.1	55.2	56.5	82.6	76.4	74.3	67.1	64.9	**61.6**	63.3	77.6	261.5	**55.8**
Jordan	37.0	103.6	183.0	106.6	79.4	84.6	**11.8**	50.5	107.2	106.2	76.1	81.3	100.4	**13.6**	232.3	**17.9**
Kazakhstan	38.8	16.2	15.9	15.9	16.5	15.7	**5.0**	50.8	15.6	16.4	**8.1**	10.1	14.2	21.9	156.9	**18.5**
Kuwait	7.1	7.0	4.5	4.7	5.6	4.8	**3.5**	22.6	7.5	19.0	11.7	5.8	**4.8**	4.9	116.8	**9.9**
Lebanon	33.1	38.0	**31.5**	38.5	38.1	38.6	94.5	33.8	**26.0**	35.2	40.8	40.9	41.6	85.2	175.7	**64.1**
Lithuania	57.9	41.0	40.6	36.4	42.2	42.2	**29.1**	41.2	55.7	94.7	127.9	55.7	56.1	**28.7**	122.3	**11.1**
Malaysia	**14.4**	14.8	22.1	22.7	24.5	23.3	29.6	35.0	28.2	23.4	25.0	25.1	25.2	**21.2**	148.1	**53.3**
Mexico	135.8	75.8	78.2	**69.0**	69.6	77.8	97.9	144.5	71.8	73.5	76.6	70.7	**66.8**	81.9	1,192.0	**165.0**
Moldova	47.6	**5.9**	5.9	6.1	6.0	6.4	6.4	46.0	6.4	6.6	20.6	6.3	7.5	**5.2**	112.4	**9.0**
Morocco	85.3	13.9	14.0	15.1	17.2	16.3	**1.3**	61.5	62.2	111.7	22.3	19.9	87.1	**7.6**	328.3	**10.6**
Nepal	90.3	15.0	15.0	49.7	42.7	41.1	**4.9**	30.8	40.4	4.4	70.5	47.2	54.5	**3.0**	190.9	**2.8**
Netherlands	83.1	102.9	42.9	**37.8**	39.0	44.1	128.8	108.4	65.7	100.9	115.8	**41.6**	42.6	140.4	-	**55.2**
Pakistan	16.6	33.3	13.8	18.0	18.2	18.1	**3.5**	**9.2**	33.6	69.3	25.4	27.7	18.2	11.7	374.1	**25.0**
Panama	28.4	33.5	23.9	23.7	23.7	**23.4**	47.7	29.9	27.4	28.9	40.2	29.0	**26.7**	271.4	214.6	**23.8**
Paraguay	13.0	3.3	3.0	2.5	**1.8**	1.8	2.8	15.8	**3.7**	3.9	4.4	10.4	8.1	10.3	86.0	**10.9**
Peru	49.3	**43.3**	43.3	43.3	43.3	43.3	53.2	228.7	71.9	162.3	144.9	308.0	**39.7**	41.5	813.8	**93.8**
Philippines	157.4	53.1	39.1	38.7	35.6	14.4	**11.9**	89.2	78.3	119.0	12.5	39.0	24.5	**11.9**	361.1	**26.9**
Poland	218.1	119.4	100.9	94.3	89.1	97.6	**63.8**	168.8	208.6	71.1	79.0	92.3	72.4	**50.8**	970.4	**78.1**
Portugal	**58.7**	78.5	80.4	80.0	79.6	80.2	268.2	**54.3**	83.0	86.5	78.4	88.4	89.1	165.1	452.8	**144.1**
Qatar	24.2	10.8	5.6	6.7	5.5	6.9	**3.8**	34.0	16.6	5.4	5.4	12.8	5.8	**3.8**	104.8	**5.0**
Romania	119.4	55.6	71.8	57.9	72.9	74.7	**18.2**	180.4	142.1	91.6	72.1	120.0	83.4	**28.8**	505.9	**34.0**
Russia	443.7	56.9	187.5	182.3	176.9	279.9	**36.5**	243.0	540.5	**56.3**	99.1	167.3	924.9	266.1	2,507.5	**249.2**
Saudi Arabia	86.6	12.2	6.5	6.3	5.8	5.5	**3.6**	49.4	10.8	16.5	13.3	9.0	5.4	**5.2**	260.8	**4.1**
Serbia	224.7	240.3	**49.6**	169.1	167.0	167.0	78.7	87.5	42.2	**24.1**	136.6	151.3	138.8	104.8	289.9	**22.5**
Slovakia	55.2	20.8	30.2	22.1	**18.0**	19.9	31.4	52.2	**17.9**	59.2	21.7	54.3	19.2	20.0	172.1	**32.1**
Slovenia	46.8	**4.4**	18.5	10.3	26.5	26.7	5.7	19.7	9.2	26.3	21.7	30.1	39.0	**5.8**	113.6	**17.8**
South Africa	239.2	187.0	56.1	63.8	64.1	**41.3**	1,138.3	222.2	175.9	64.6	**52.0**	65.5	64.5	1,639.5	988.8	**76.2**
Spain	648.6	86.1	82.6	81.7	81.3	**81.2**	449.0	568.1	266.8	82.2	**72.8**	84.7	85.0	162.7	-	**379.2**
Sweden	57.0	48.4	46.1	46.2	**46.0**	46.2	133.9	69.8	**21.3**	44.6	43.1	57.3	56.6	185.9	-	**42.9**
Switzerland	88.1	84.0	83.2	84.1	84.5	83.2	**18.5**	66.7	91.6	88.2	82.8	90.6	91.6	**15.1**	274.1	**22.8**
Tunisia	22.6	**19.5**	19.5	19.7	20.5	20.4	27.4	24.0	24.2	**22.4**	23.2	23.2	22.9	56.4	130.7	**26.4**
Turkey	220.4	287.0	227.8	418.9	461.3	476.3	**144.4**	247.4	241.3	209.1	619.6	479.1	509.8	**177.2**	1,741.9	**98.4**
UAE	79.8	50.0	27.4	19.0	20.8	**18.6**	51.4	18.9	47.6	20.0	19.3	**14.6**	37.1	71.9	224.1	**52.8**
UK	**236.7**	363.8	510.5	432.3	360.7	355.7	821.7	**236.3**	606.8	576.0	785.8	457.7	639.5	963.0	-	**377.8**
Ukraine	287.4	130.7	105.0	109.9	103.2	108.5	**80.2**	373.9	186.8	220.0	150.2	106.4	196.2	**86.2**	813.1	**57.6**
US	137.8	144.7	134.1	134.4	132.0	**90.0**	134.9	139.9	117.1	148.0	196.2	160.6	123.7	**114.8**	-	**100.3**
No. Rank 1	6	9	6	4	5	8	**32**	5	6	6	7	6	7	**33**	0	**70**

Note that with segmentation, only the last segment, not the entire sequence, is used for prediction. Despite the fact, segmentation increases the accuracy of prediction by letting epidemic models focus on the part that represents the current epidemic dynamics while ignoring the part before inherent changes in the dynamics.

### 5.5 Additional experimental results

Below, we present the results of additional experiments.

#### 5.5.1 Insensitivity to two arguments: xtol and ftol

For optimization, we used the lmfit library provided in Python, which minimizes non-linear least-squares. The leastsq function, which we used, requires two arguments, xtol and ftol, which are the desired relative errors in the approximation solution and the sum-of-squares, respectively (see https://lmfit.github.io/lmfit-py/fitting.html#lmfit.minimizer.Minimizer.leastsq for details.). We tested the NLLD model in the Japan dataset using eight different xtol and ftol values (10^−1^ to 10^−8^) and five different latent factors *k* (2 to 6). In the 40 considered settings, the splitting points of the segmentation were exactly the same (71^th^, 198^th^, and 324^th^ day), which implies that the proposed scheme is insensitive to these parameters. Thus, in this work, we do not tune xtol and ftol but fix them to 10^−8^ in all experiments in the main paper.

#### 5.5.2 The effect of the constraint on the last segment

**Fig 6:**
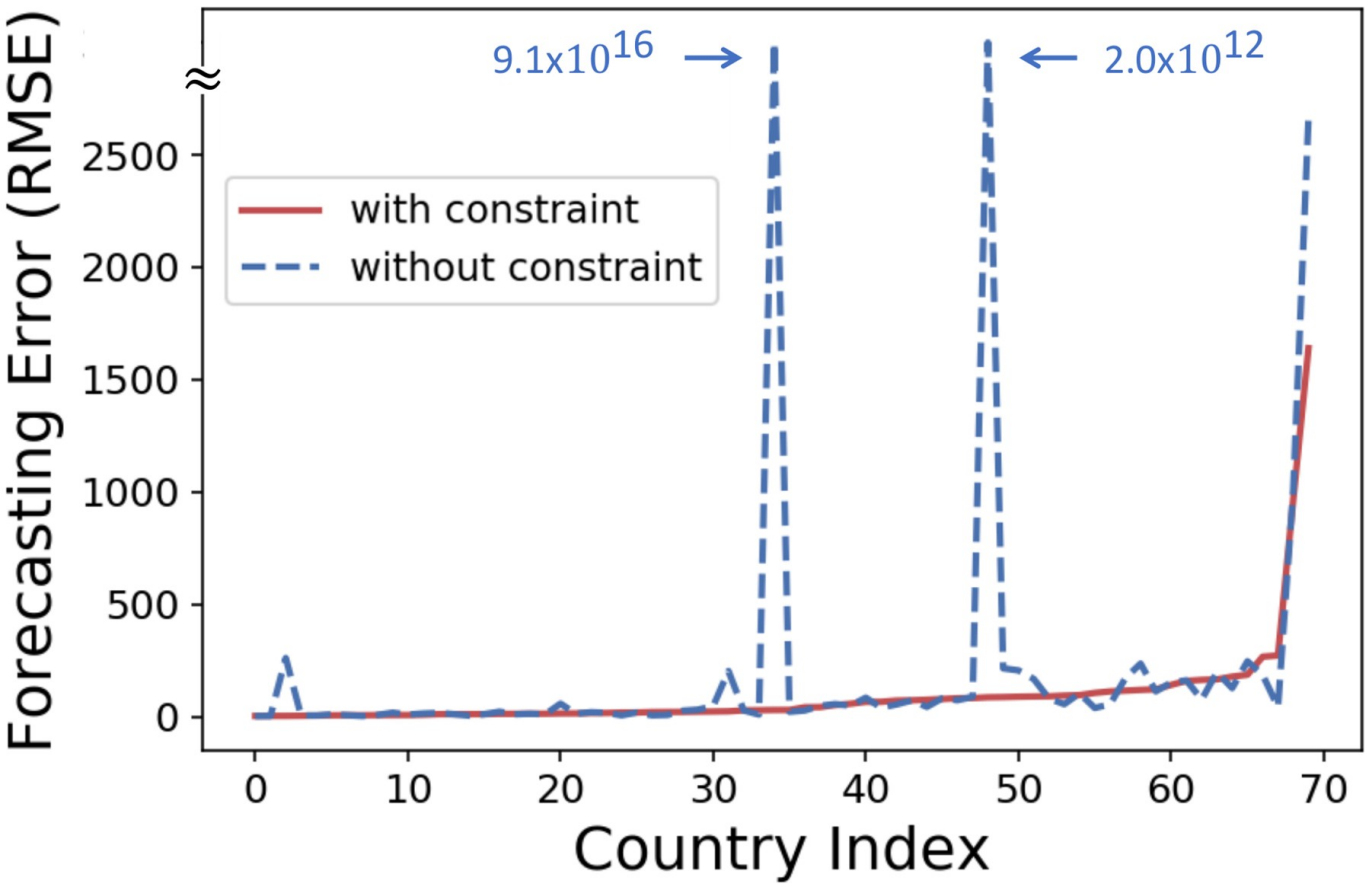
Ensuring the length of the last segment in the training set to 37 days is helpful for accurate prediction. NLLD without the constraint sometimes greatly overestimates the numbers of infected and recovered individuals, and the constraint helps prevent such absurd predictions. Moreover, the constraint was beneficial in 39 countries out of the 70 countries. The countries are indexed in the order of the forecasting error of NLLD with the constraint.

One might concern that avoiding segmentation within the last 37 days before the test set may degrade the flexibility of the model and thus the accuracy of forecasting. Empirically, however, this constraint is helpful for accurate prediction by preventing overfitting. Note that if the length of the last segment is too short, overfitting easily occurs, resulting in a large generalization (i.e., prediction) error. In order to demonstrate the effect of the constraint, we compared the forecasting errors of the NLLD model with (our original setting) and without the constraint in 70 countries. As shown in Fig 6, without the constraint, NLLD greatly overestimated the numbers of infected and recovered individuals in some countries (specifically, Lebanon and Lithuania). It should be noted that the estimates were even larger than the population of the countries. On the other hand, the constraint helped preventing such absurd predictions, and specifically, NLLD with the constraint always made predictions within the population of the countries. In addition, out of the 70 countries, NLLD with the constraint outperformed that without the constraint in 39 countries. The average forecasting error (in terms of RMSE) was also smaller when adopting the constraint. Specifically, it was 94.3 with the constraint and 116.3 without the constraint (averaged only the reasonable results in the 68 countries).

## 6 Conclusions

In this work, we propose to divide epidemic event sequences into multiple segments and fit a simple model to each segment. To this end, we propose a greedy algorithm based on the MDL principle to decide where to split the sequences. Through extensive experiments using the COVID-19 event sequences from 70 countries, we demonstrate that our methodology has the following advantages:

- **Automatic**: All parameters are tuned automatically based on the MDL principle without relying on users.
- **Model-agnostic**: Any ODE-based epidemic models can be used with our segmentation scheme.
- **Effective**: The fitting error and prediction error of three epidemic models decrease up to 14.29× and 31.54×, respectively, with our segmentation scheme.

**Reproducibility**: The code and datasets used in the paper are available at https://github.com/geonlee0325/covid_segmentation.

## Supporting information

S1 Appendix (PDF)
