# Supplementary material for: Simple epidemic models with segmentation can be better than complex ones
Source: PLoS One. 2022 Jan 12;17(1):e0262244. doi: 10.1371/journal.pone.0262244 (PMC8754337; doi:10.1371/journal.pone.0262244)
Supplement: S1 Appendix — (PDF) [file pone.0262244.s001.pdf]

Simple Epidemic Models with Segmentation Can Be Better than Complex Ones (SUPPLEMENTARY DOCUMENT)

Geon Lee[a], Se-eun Yoon[b], Kijung Shin[a,b*]

[a] *Graduate School of AI, KAIST, Daejeon, South Korea*
[b] *School of Electrical Engineering, KAIST, Daejeon, South Korea*

Abstract

In this supplementary document, we provide additional experimental results that are not included in the main paper, which is titled *Simple Epidemic Models with Segmentation Can Be Better than Complex Ones*.

Keywords: COVID-19, Epidemic models, Segmentation, Minimum Description Length

Figure 1. In Figure 1, we provide the input and estimated event sequences when the NLLD model is used with our proposed segmentation scheme.
Figure 2, 3, and 4. We demonstrate the effectiveness by measuring how segmentation affects the model complexity and fitting error of the three considered epidemic models. We provide the results when the NLLD, LLD, and SIR models are used in Figure 2, Figure 3, and Figure 4, respectively.
Figure 5, 6, and 7. In addition, we examine the effectiveness of our greedy segmentation scheme based on the MDL principle by comparing it with the incremental segmentation method, in terms of the model complexity and fitting error of the three considered epidemic models. We provide the results when the NLLD, LLD, and SIR models are used in Figure 5, Figure 6, and Figure 7, respectively.

*Corresponding author
Email addresses: geonlee0325@kaist.ac.kr (Geon Lee), granelle@kaist.ac.kr (Se-eun Yoon), kijungs@kaist.ac.kr (Kijung Shin)

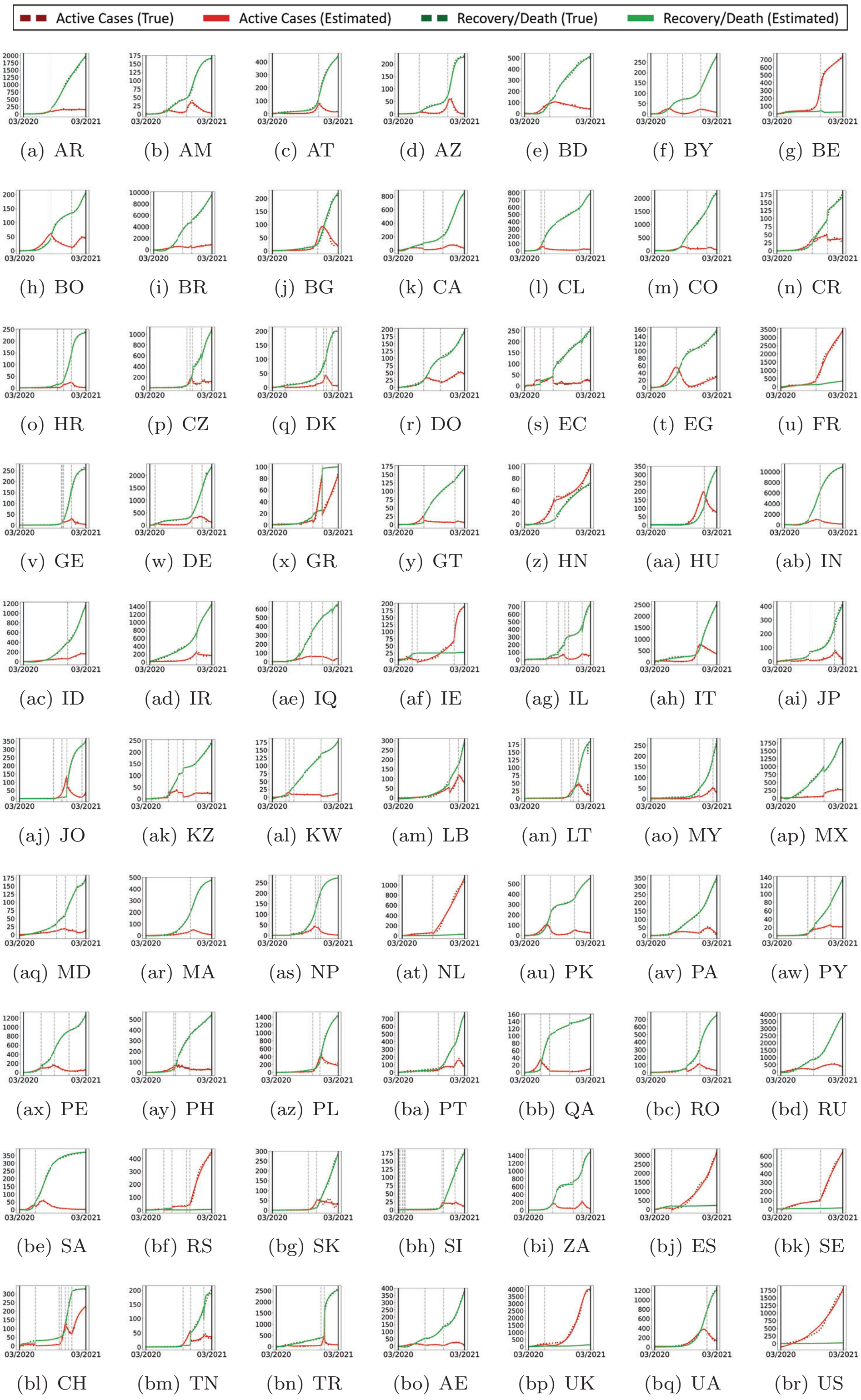

Figure 1: The true and estimated event sequences when the NLLDmodel is used with our proposed segmentation scheme. The scale of the values in the y-axis are thousands ($10^3\times$).

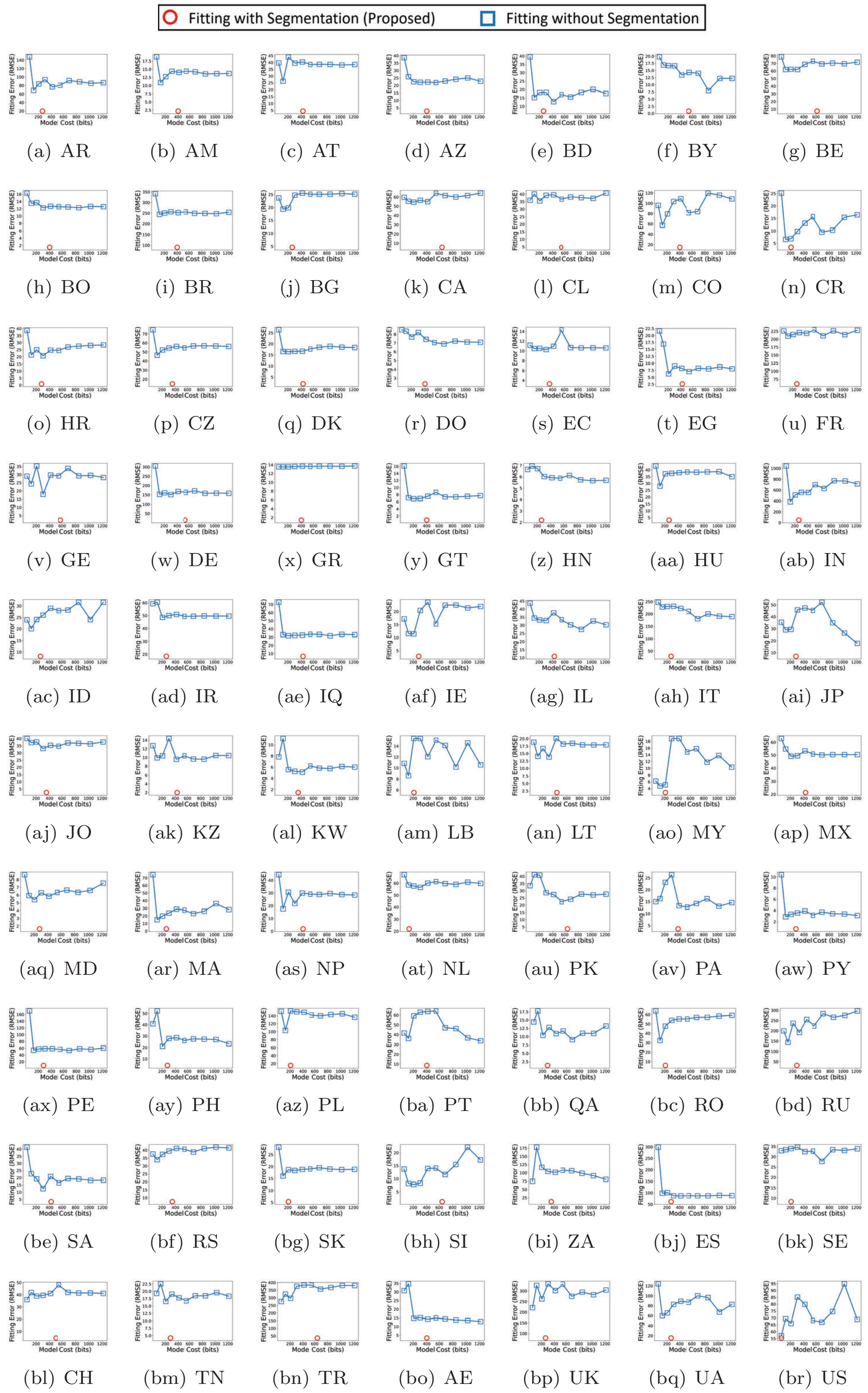

Figure 2: The true and estimated event sequences when the NLLDmodel is used with our proposed segmentation scheme. The scale of the values in the y-axis are thousands ($10^3\times$).

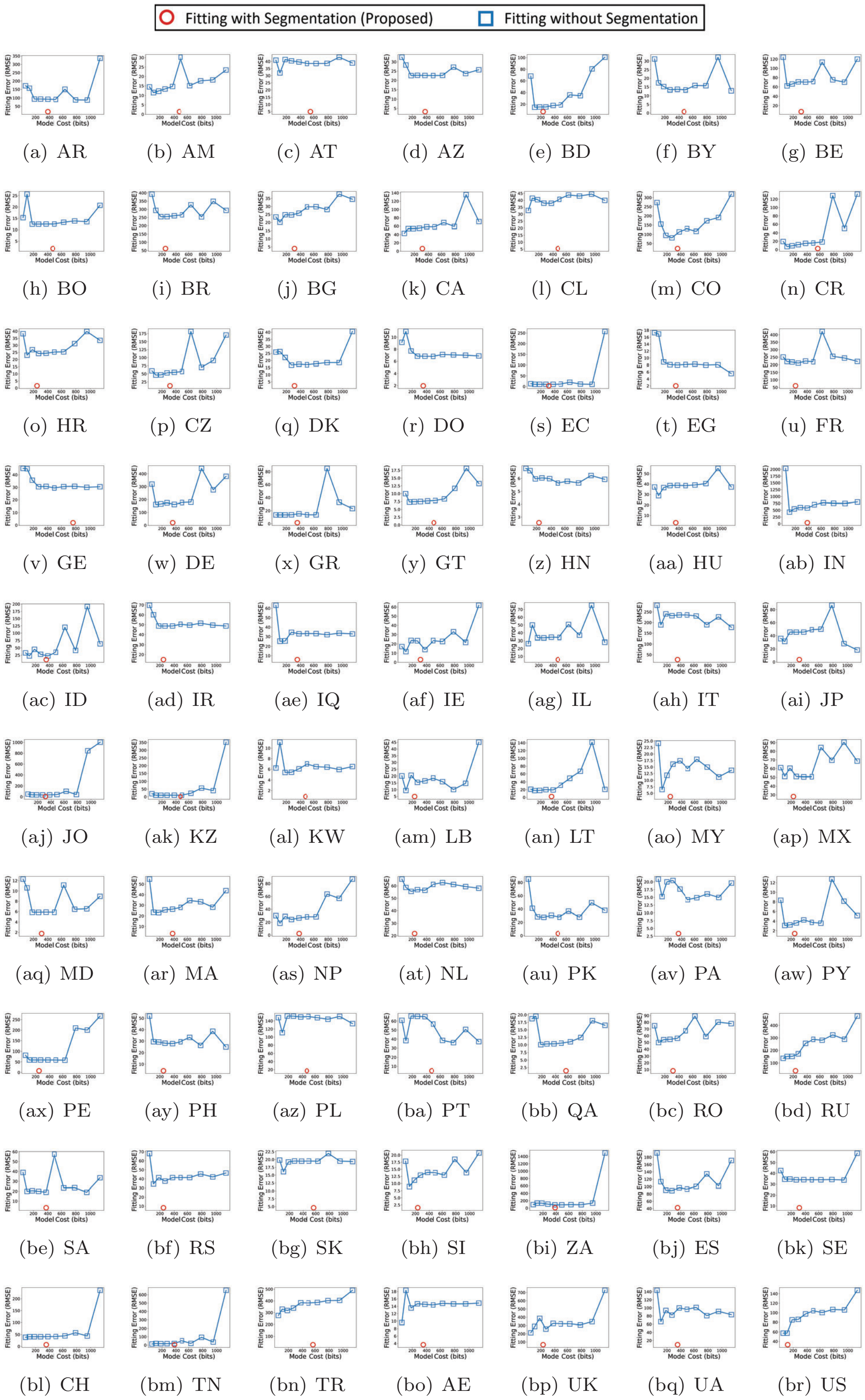

Figure 3: The true and estimated event sequences when the LLDmodel is used with our proposed segmentation scheme. The scale of the values in the y-axis are thousands ($10^3\times$).

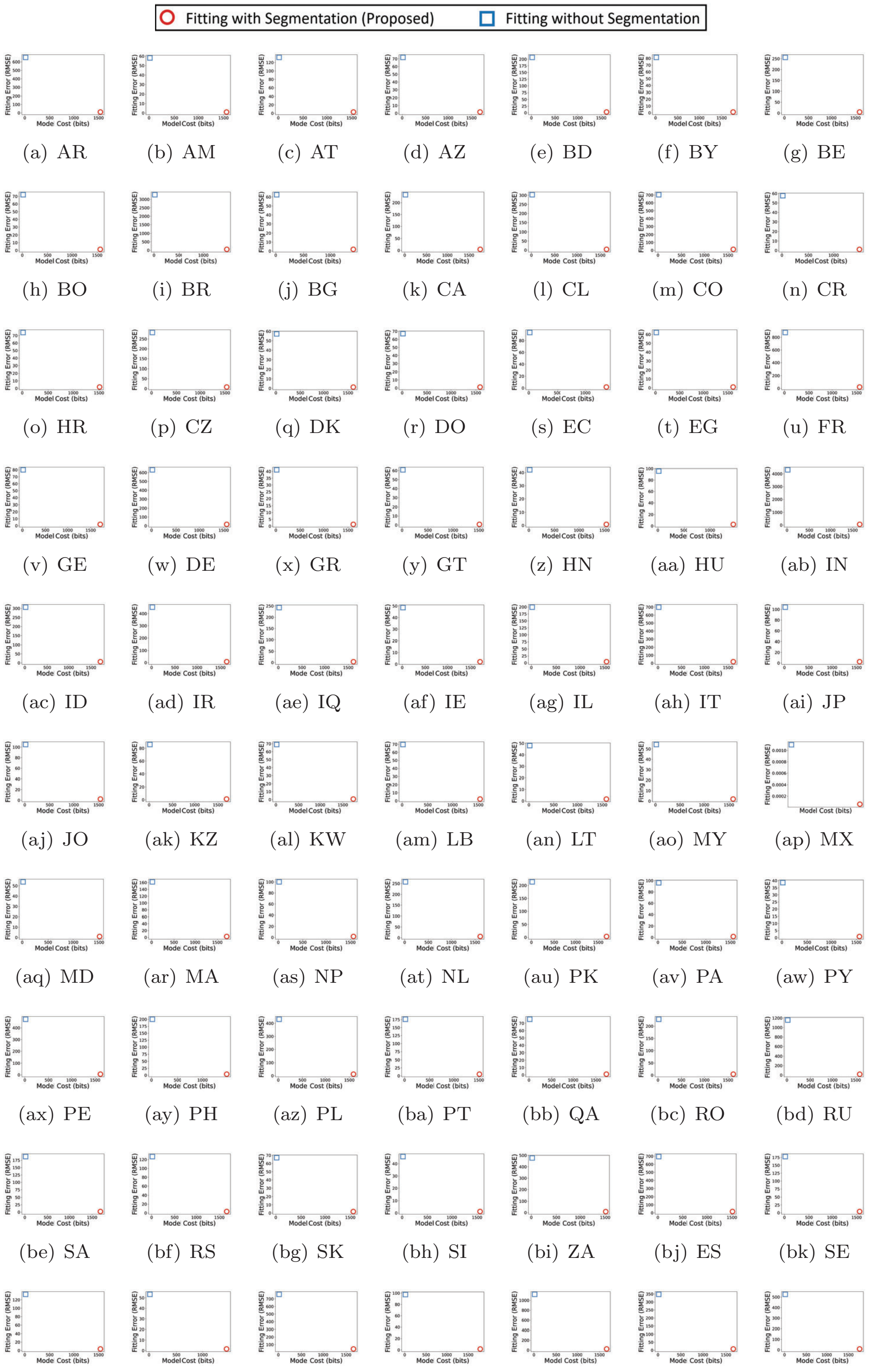

(a) AR (b) AM (c) AT (d) AZ (e) BD (f) BY (g) BE

(h) BO (i) BR (j) BG (k) CA (l) CL (m) CO (n) CR

(o) HR (p) CZ (q) DK (r) DO (s) EC (t) EG (u) FR

(v) GE (w) DE (x) GR (y) GT (z) HN (aa) HU (ab) IN

(ac) ID (ad) IR (ae) IQ (af) IE (ag) IL (ah) IT (ai) JP

(aj) JO (ak) KZ (al) KW (am) LB (an) LT (ao) MY (ap) MX

(aq) MD (ar) MA (as) NP (at) NL (au) PK (av) PA (aw) PY

(ax) PE (ay) PH (az) PL (ba) PT (bb) QA (bc) RO (bd) RU

(be) SA (bf) RS (bg) SK (bh) SI (bi) ZA (bj) ES (bk) SE

(bl) CH (bm) TN (bn) TR (bo) AE (bp) UK (bq) UA (br) US

Figure 4: The true and estimated event sequences when the SIR model is used with our proposed segmentation scheme. The scale of the values in the y-axis are thousands ($10^3\times$).

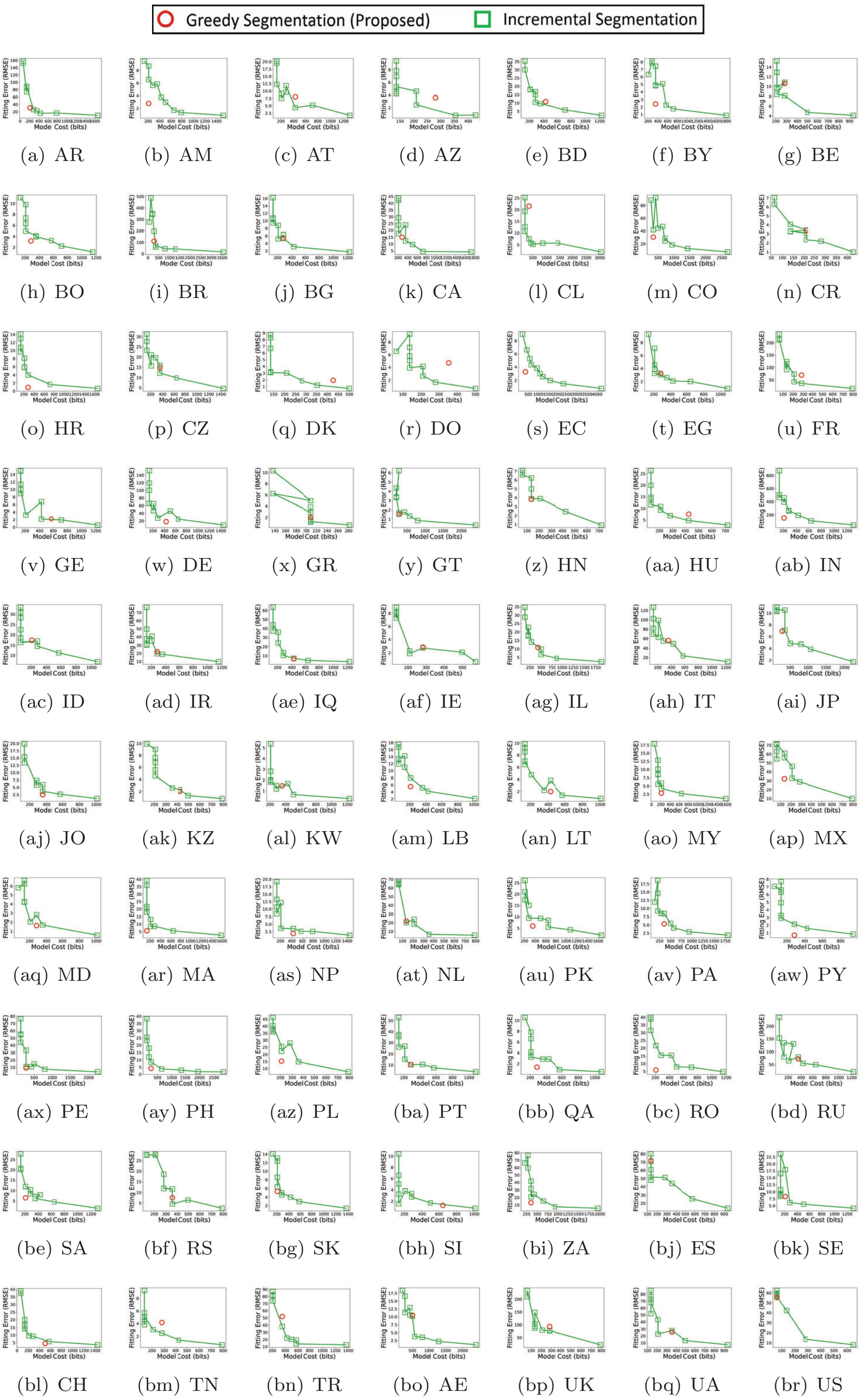

Figure 5: The true and estimated event sequences when the NLLDmodel is used with our proposed segmentation scheme. The scale of the values in the y-axis are thousands ($10^3\times$).

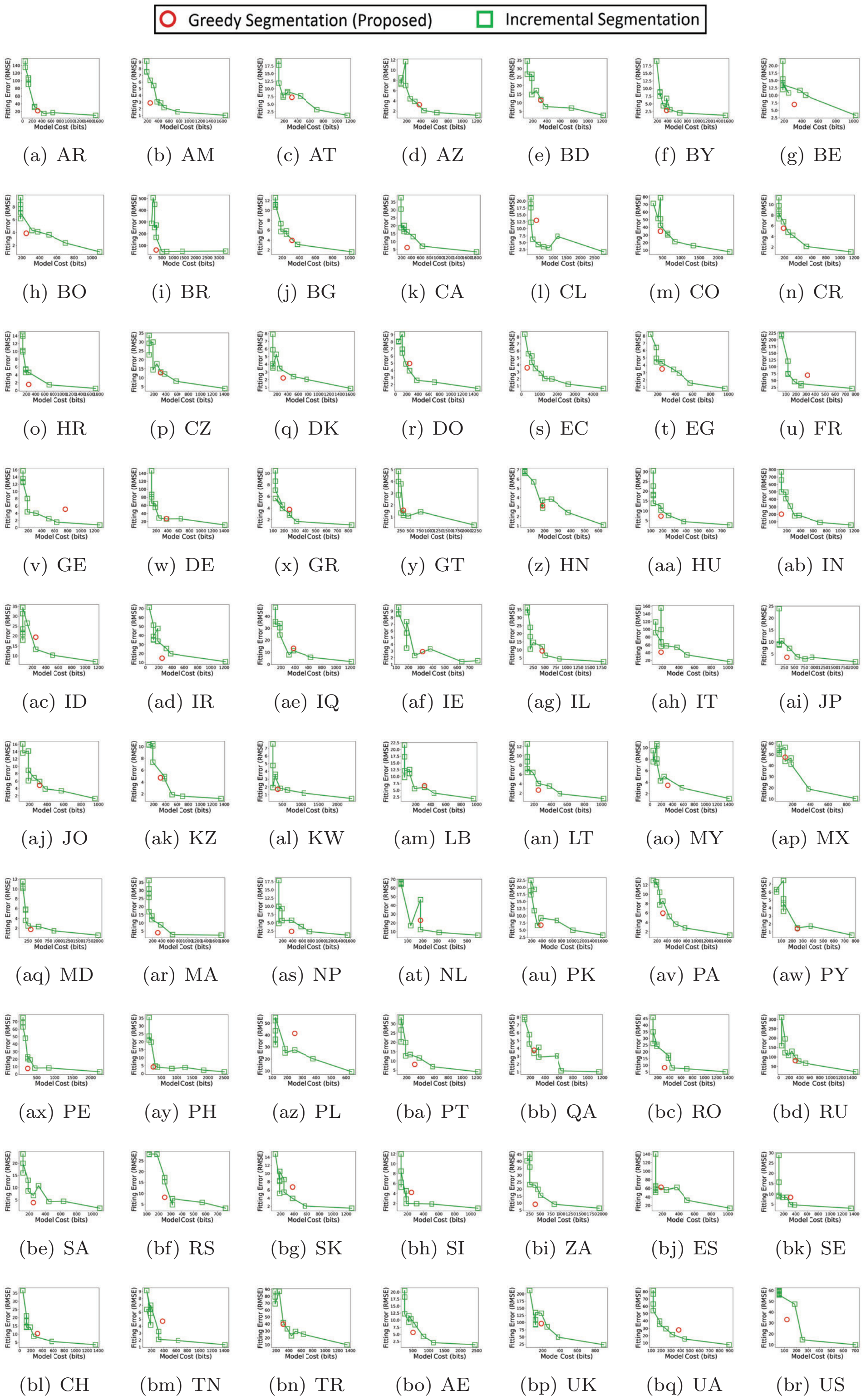

Figure 6: The true and estimated event sequences when the LLDmodel is used with our proposed segmentation scheme. The scale of the values in the y-axis are thousands ($10^3\times$).

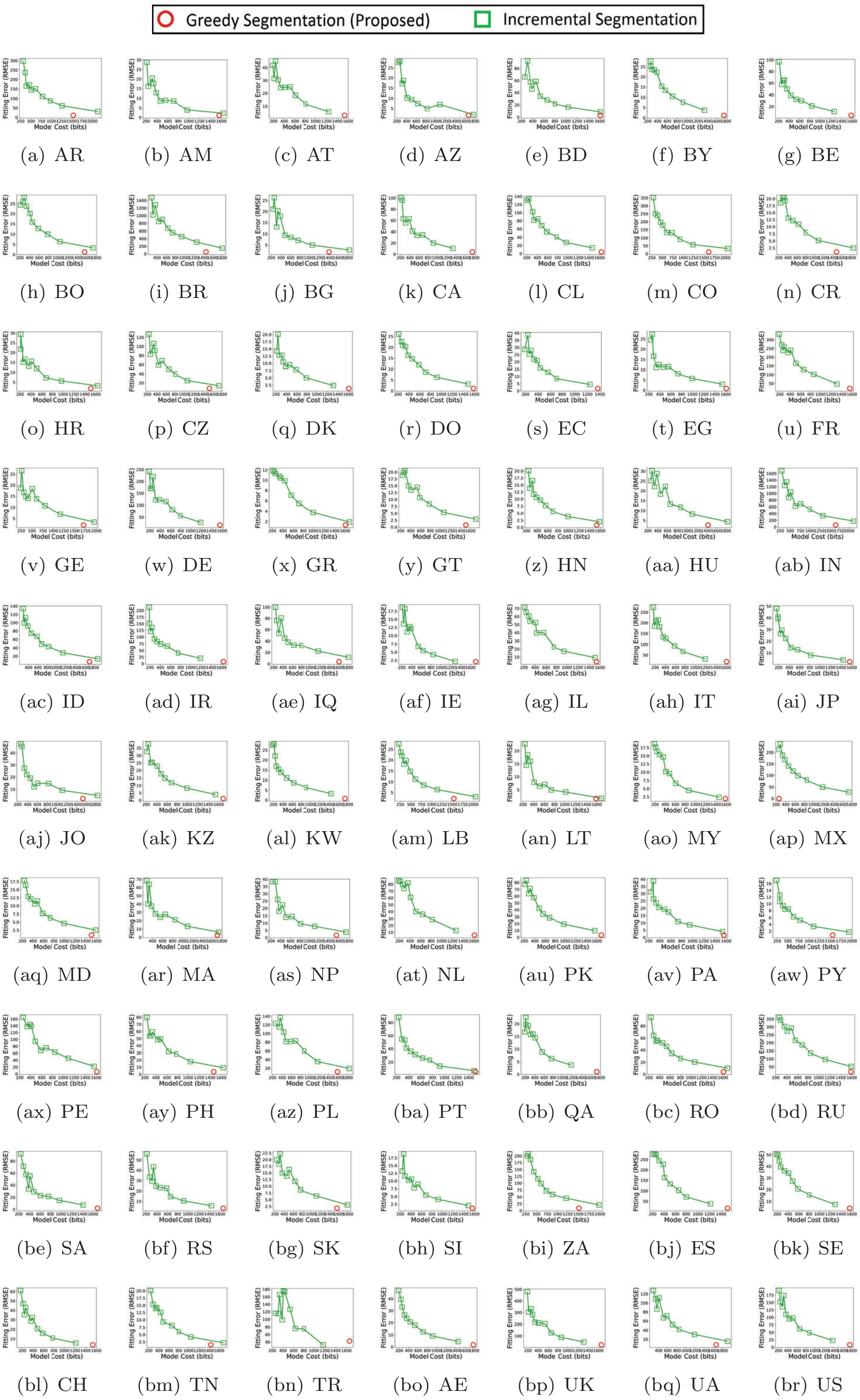

Figure 7: The true and estimated event sequences when the SIR model is used with our proposed segmentation scheme. The scale of the values in the y-axis are thousands ($10^3\times$).
